# Toxicity of *Crepis lacera* in grazing ruminants

**DOI:** 10.1186/s12917-018-1393-4

**Published:** 2018-03-07

**Authors:** Rosario Russo, Brunella Restucci, Antonio Vassallo, Laura Cortese, Massimiliano D’Ambola, Serena Montagnaro, Roberto Ciarcia, Salvatore Florio, Nunziatina De Tommasi, Lorella Severino

**Affiliations:** 1 Dipartimento di Medicina Veterinaria e Produzioni Animali, Università degli Studi di Napoli Federico II, via Delpino 1, 80137 Naples, Italy; 2 Dipartimento di Scienze, Università di Basilicata, Viale dell’Ateneo Lucano, 85100 Potenza, Italy; 3 Dipartimento di Farmacia, Università di Salerno, Via Giovanni Paolo II 132, 84084 Fisciano, SA Italy

**Keywords:** *Crepis lacera*, MDBK cells, In vivo and in vitro study, Sheep

## Abstract

**Background:**

*Crepis lacera* is a plant from the Asteraceae family that is common in the Mediterranean region. Farmers believe that this plant may be deadly to small ruminants in areas of southern Italy. However, scientific evidence is lacking, and no proof exists that *C. lacera* is toxic to ruminants. Necropsies conducted on four sheep revealed lesions in their livers and kidneys.

**Results:**

In the current study, we described sheep poisoning and isolated secondary metabolites from *Crepis lacera* to assess the metabolites’ biological activity both in vitro and in vivo. Phytochemical study of the aerial portions of *Crepis lacera* led to the isolation of five sesquiterpene lactones and two phenolic compounds. Cellular viability was evaluated in cell cultures of the bovine kidney cell line *Madin Darby Bovine Kidney* (MDBK) after incubation with phytochemicals. Our results showed that three sesquiterpene lactones, 8-epidesacylcynaropicrin-3-O-β-glucopyranoside (**2**), 8-epigrosheimin (**3**), and 8-β-hydroxydehydrozaluzanin C (**4**), were cytotoxic after 48 h of incubation. In addition, in the in vivo study, animals that received 1 mg/kg body weight (bw) of *Crepis lacera* extract and were then sacrificed after 48 h showed significant lesions in their liver, lungs and kidneys. These lesions were also found in rats that received 2 mg/kg bw of the same extract and sacrificed after 24 and 48 h.

**Conclusions:**

These results validate the hypothesis that *C. lacera* is potentially dangerous when ingested in large quantities by grazing small domestic ruminants. Further studies are necessary to clarify the molecular mechanisms of *Crepis spp.* toxicity in animals.

## Background

*Crepis lacera* is a plant in the Asteraceae family that is commonly found in many areas of central and southern Italy at 700 to 1200 m altitudes. It is a 15 to 40-cm tall perennial herb with erect stems branching from the upper section. *C. lacera* has nutritional properties common to many bitter herbs, such as detoxification, purification, diuretic and hypoglycemic effects [1]. Nevertheless, many Italian farmers believe that this plant is fatal if frequently ingested by ruminants such as sheep and cattle, in areas of southern Italy during late spring and summer when *C. lacera* grows copiously.

To date, scientific evidence of *Crepis lacera* toxicity in ruminants is lacking, and no toxic compound has yet been identified. The current study started with an on-site investigation, during which we found two deceased and two dying sheep. We necropsied animals, that, per the farmers reports, had died after consuming the plant. Plant specimens were collected, and a phytochemical study was conducted to isolate and characterize *Crepis lacera* secondary metabolites. In addition, we investigated the toxic effects of these metabolites using both in vitro and in vivo models. Considering the injuries to the kidneys found during necropsy, the biological activity of the isolated compounds was evaluated in cell cultures of *Madin Darby Bovine Kidney* (MDBK) cells. Finally, the crude extract was fed to rats via gastro-esophageal gavage to assess toxicity in vivo.

## Methods

### History

Veterinary practitioners from different farms located in the Basilicata region in southern Italy contacted the division of Veterinary Toxicology of the Department of Veterinary Medicine and Animal Productions of University Federico II of Naples, reporting that many sheep suddenly died after grazing in the pasture during late spring and throughout the summer. Food poisoning was suspected. Veterinarians also reported that such fatalities were recurrent episodes and that every summer, several farm animals died under similar circumstances.

During the on-site visit, we found two dead sheep in the pasture. The other two sheep were permanently in lateral recumbency and were clinically examined, with attention to the cardiovascular apparatus. Standard 6-lead electrocardiograms (ECGs), including bipolar and augmented limb leads (I, II, III, aVR, aVL, aVF), were performed using a single-channel electrocardiograph (08SD, BTL Italia, Italy). The electrodes were connected to the medial side skin of the elbows and stifles, using alligator-type electrodes attached to the skin. The paper speed was 25 mm/s, and the electrocardiogram was calibrated at 1 mV = 10 mm. A 2-min strip was recorded per sheep, and ECGs were recorded. Heart rate was calculated by averaging six R–R intervals (in lead-1), and ECGs were evaluated to determine features suggestive of abnormalities. It was impossible to perform urinalysis, hematology and serum biochemistry because the sheep died shortly after clinical examination.

### Necropsy

Necropsies were performed on the 4 sheep, two of which died after clinical examination, and two were found dead on the pasture. Samples from all organs, collected immediately after necropsy, were fixed in 10% neutral buffered formalin and routinely processed at the Veterinary Pathology division. Paraffinized sections were cut at 4 μm and stained with hematoxylin and eosin.

### Phytochemical study

#### Plant material

*Crepis lacera* (the aerial portion) was collected from public pastures in the Basilicata region (southern Italy) from May to July 2010, and the sampling was repeated annually over the next 4 years.

#### General experimental procedures

Optical rotations were measured on a Rudolph Research Analytical Autopol IV polarimeter equipped with a sodium lamp (589 nm) and a 1-dm microcell. NMR experiments were performed on a Bruker DRX-600 spectrometer (Bruker BioSpin GmBH, Rheinstetten, Germany) equipped with a Bruker 5-mm TCI CryoProbe at 300 K. All 2D NMR spectra were acquired in methanol-*d*_*4*_ (99.95%, Sigma-Aldrich, St. Louis, MO, USA), and standard pulse sequences and phase cycling were used for DQF-COSY, HSQC, and HMBC spectra. ESI-MS were obtained using a Finnigan LC-Q Advantage Thermoquest spectrometer, equipped with Xcalibur software. HR-ESIMS spectra were acquired in positive ion mode on a Q-TOF premier spectrometer (Waters, Milford, Massachusetts, USA). TLC was performed on pre-coated Kieselgel 60 F_254_ plates (Merck KGaA, Darmstadt, Germany); compounds were detected by spraying with Ce(SO_4_)_2_/H_2_SO_4_ solution. Column chromatography was performed over silica gel (70–220 mesh, Merck); reversed-phase (RP) HPLC separations were conducted on a Shimadzu LC-20AT series pumping system equipped with a Shimadzu RID10A refractive index detector and a Shimadzu injector.

#### Extraction and isolation of compounds

Aerial portions of *Crepis lacera* (500 g) were defatted with *n*-hexane and successively extracted with CHCl_3_; CHCl_3_-MeOH (9:1) and MeOH by exhaustive maceration (3 × 2 L), yielding 14.0, 10.0, 5.0 and 35.0 g of the residues. Five grams of the CHCl_3_-MeOH (9:1) extract was chromatographed on a Sephadex LH-20 column, using MeOH as the eluent. Fifty fractions were collected (8 ml each) and grouped based on TLC results in six fractions (A-F). Fraction B (1 mg) was purified by RP-HPLC using a C_18_ μ-Bondapak column (7.8 × 300 mm, flow rate 2.0 ml min^− 1^) with MeOH-H_2_O (25:75) as the eluent to yield compounds **1** (10 mg t_R_ = 12.5 min) and **2** (8 mg t_R_ = 15.0 min). Fraction C (0.4 g) was purified by RP-HPLC using a C_18_ μ-Bondapak column (7.8 × 300 mm, flow rate 2.0 ml min^− 1^) with MeOH-H_2_O (8:17) as the eluent to yield compound **3** (15 mg t_R_ = 42.5 min). Fraction D (1.2 g) was purified by RP-HPLC using a C_18_ μ-Bondapak column (7.8 × 300 mm, flow rate 2.0 ml min^− 1^) with MeOH-H_2_O (15:35) as the eluent to yield compounds **4** (15 mg t_R_ = 16.0 min), **5** (5 mg t_R_ = 7.5 min) and **6** (17 mg t_R_ = 11.0 min). Fraction E (80 mg) was purified using a C_18_ μ-Bondapak column (7.8 × 300 mm, flow rate 2.0 ml min^− 1^) with MeOH-H_2_O (35:65) as the eluent to yield compound **7** (5 mg t_R_ = 27.5 min).

### In vitro study

#### Cell culture

*Madin Darby Bovine Kidney* (MDBK) cells (American Type Culture Collection, Rockville, MD, USA) were grown in adhesion on petri dishes and cultured in Dulbecco’s Modified Eagle’s Medium (DMEM) supplemented with 10% fetal calf serum (FCS), 2 mM L-glutamine, 100 U/ml penicillin, 100 μg/ml streptomycin, and 0.2 mM Na pyruvate at 37 °C and 5% CO_2_.

#### Cytotoxicity assay

Seven purified compounds isolated from *Crepis lacera* were dissolved in DMSO to obtain a stock solution of 5 mM. Cells (3.5 × 10^4^ cells/well) were instilled in 96-well microtiter plates and allowed to adhere for 3 h. The medium was then replaced, and 100 μl of a suspension containing 90 μl of fresh medium and 10 μl of each compound at different concentrations (50–150 μM) was added to each well. Cells were incubated for 24 and 48 h. Cell viability was assessed by an MTT colorimetric assay [2]. Briefly, 25 μl of MTT (5 mg/ml) was added, and cells were incubated for 3 h. Cells were then lysed, and the dark blue crystals were solubilized with 100 μl of a solution containing 50% (v:v) N,N-dimethylformamide and 20% (w:v) SDS with an adjusted pH of 4.5. The optical density (OD) of each well was measured with a microplate spectrophotometer (Titertek Multiskan MCC/340) equipped with a 620-nm filter.

Cell viability in response to treatment with each compound was calculated as: % viable cells = (OD treated cells/OD control cells) × 100.

### In vivo study

#### Ethics statement

The current study was carried out in accordance with the recommendations in the Guide for the Care and Use of Laboratory Animals of the National Institutes of Health. All procedure complied with current Italian and European laws (National law: D.L. 26/2014; European law: Directive 2010/63/EU).

#### Animals

Adult male Sprague-Dawley rats were obtained from Harlan Laboratories Srl (San Pietro al Natisone, Udine, Italy). Eighteen rats, weighing between 200 and 230 g and age 55 days were housed under stable environmental conditions (temperature at 22 ± 2 °C and a 12-h light-dark cycle; relative humidity of 40% – 70%, artificial illumination on a 12 h light/dark cycle, and air exchange of 15 times/h.). Rats were fed a standard diet with access to food and water ad libitum.

The number of animals was calculated with GPower Sofware by using Student t-test with *P* < 0.05 and power (1-β) = 0.80.

#### Experimental in vivo procedures

Rats were randomized into three groups (6 animals per group) by treatment. Considering the results from the phytochemical study, we used the CHCl_3_-MeOH (9:1) extract for the in vivo study. The extract was solubilized with castor oil (including 10% DMSO to minimize any undesired effects of the vehicle).

The first group was treated with 1 ml of *C. lacera* extract at 1 mg/kg body weight (bw) daily by intragastric gavage at 24 and 48 h. The second group was treated with 1 ml of *C. lacera* extract at 2 mg/kg bw daily by intragastric gavage at 24 and 48 h. The control group received a daily equivalent volume of the vehicle.

Twenty-four and 48 h after ingesting either the *C. lacera* extract or the vehicle, animals were weighed and sacrificed under anesthesia by an intraperitoneal injection of Inactin (Sigma-Aldrich, St. Louis, MO, USA), 120 mg/kg bw. Necropsies were performed and the liver, kidneys and lungs were collected from all animals. Tissue samples were fixed in 10% neutral buffered formalin and routinely processed. Sections (4 μm) were stained with hematoxylin and eosin for histological examination.

#### Statistical analysis

Data (from the in vitro study) represent the mean ± standard deviation (SD) of three independent experiments. All values were statistically analyzed using Student’s t-test. The significance level was set at *P* < 0.05.

## Results

When we clinically examined the dying sheep, they were depressed and in lateral recumbency. No abnormal sounds were heard upon auscultating the lungs and heart. The ECG recordings showed no electrocardiographic features suggestive of myocardial hypoxia/ischemia. No sheep had sinus tachycardia, and the heart rates were 45 beats per minute in one sheep and 60 in the other. Both sheep showed bradycardia, and sporadic ventricular premature complexes (VPCs) were recorded in one sheep. No other clinical signs were observed, and these sheep died shortly after.

Post-mortem examinations on the four sheep carcasses revealed severe lesions in both their kidneys and livers. The lesions were similar in both the sheep found dead and in those that died shortly after clinical examination. The kidneys were pale and enlarged and showed disseminated and petechial hemorrhages, which were also observed on the cut surface. The renal medulla was reddish. The liver was enlarged and showed rounded edges and a pale surface with multifocal petechial hemorrhages. Small patchy pale and soft foci were observed on the surface as well as on deep parenchymal sections. Diffuse hemorrhages were found in the abomasum and intestinal mucosa. The myocardium was pale and dry with small hemorrhages on the epicardial surface of the interventricular septum. Bilateral pulmonary edema was also present.

**Fig. 1:**
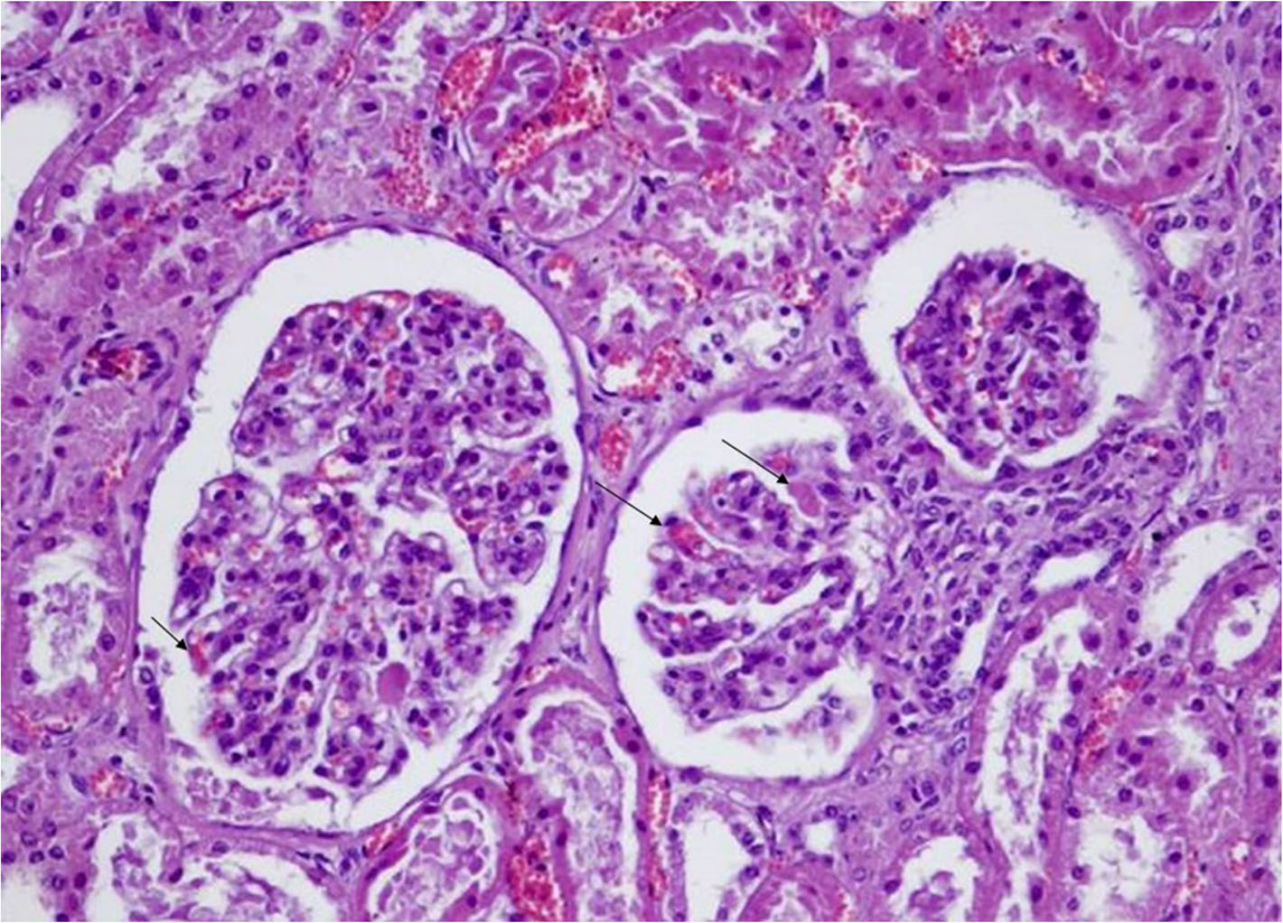
Sheep kidney: shrunken glomeruli and others modified in shape with small hyaline material deposits mixed with erythrocytes (arrows) into the glomerular vessels, numerous small hemorrhages and proximal tubular acute necrosis are evident. Hematoxylin-eosin, optical microscopy 20×

**Fig. 2:**
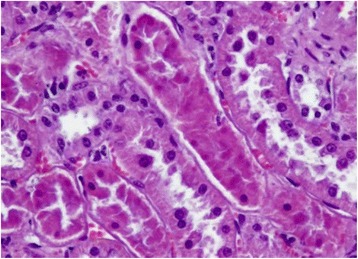
Sheep kidney: Numerous tubules show epithelial cell necrosis characterized by eosinophilic amorphous cytoplasm and nuclear loss. Hematoxylin-eosin, optical microscopy 40×

**Fig. 3:**
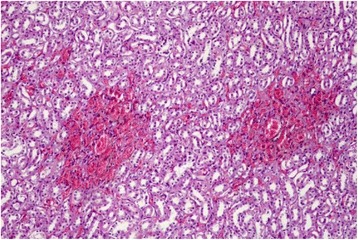
Sheep kidney: multifocal perivascular hemorrhages among tubules in the medulla are evident. Hematoxylin-eosin, optical microscopy 20×

**Fig. 4:**
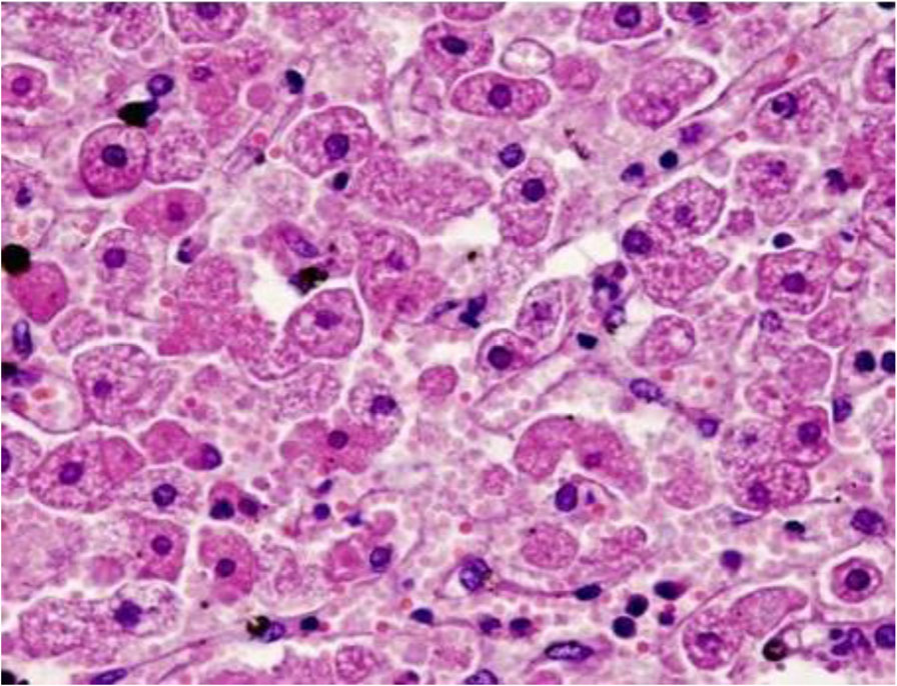
Sheep liver: Extensive necrosis resulting in collapsed hepatic cords and individualized hepatocytes showing degenerative alterations characterized by little and multiple cytoplasmic vacuoles and karyopicnosis are evident. Hematoxylin-eosin, optical microscopy 40×

Histological examination of the kidneys revealed several shrunken glomeruli and others that were modified in shape with small hyaline material deposits in the glomerular vessels (Fig. 1). The most severe lesions were coagulate necrosis of the tubular epithelial cells characterized by cytoplasmic eosinophilia and nuclear karyolysis, as well as by loss of nuclei, in both the distal and proximal renal tubules (Fig. 2). Medullary areas contained multifocal perivascular hemorrhages (Fig. 3). Liver tissue showed entire lobules with degenerative hepatocyte changes, which appeared swollen, containing multiple, small eosinophilic vacuoles in the cytoplasm, karyolysis and karyopicnosis. Multifocal areas of coagulation necrosis resulting in collapsed hepatic cords and hepatocyte loss were scattered throughout, but chiefly in the centrolobular zone. Few lymphocytes and plasma cells were observed near the necrotic foci (Fig. 4).

**Fig. 5:**
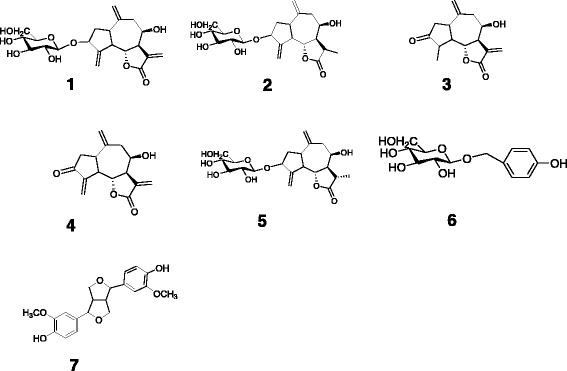
Compounds (**1**–**7**) isolated from *Crepis lacera* aerial portions extract

The histological features strongly indicated acute hepato- and nephrotoxicosis. The toxicity of all extracts obtained from the *C. lacera* aerial portions (0.5–2.5 mg/ml) was assessed in a preliminary in vitro study using MDBK cells. We found that chloroform-methanol (9:1) extract was cytotoxic after 48 h of incubation (data not shown). Therefore, chloroform-methanol (9:1) extract was chromatographically analyzed on Sephadex LH-20 columns, and six fractions (A-F) were collected. Each fraction was evaluated individually for cytotoxicity (data not shown). Fractions B-E were the main toxic components of the chloroform-methanol (9:1) extract; thus, fractions B-E were separated by RP-HPLC, yielding seven known compounds identified as follows: crepiside D (**1**) [3],8-epidesacylcynaropicrin-3-*O*-*β*-glucopyranoside (**2**) [4], 8-epigrosheimin (**3**) [5], 8-*β*-hydroxydehydrozaluzanin C (**4**) [6], 11-dehydrocrepiside D (**5)** [3], *p-*hydroxy-benzyl 7-O- β-glucopyranoside (**6**) [6], and pynoresynol (**7**) [7] (Fig. 5).

**Fig. 6:**
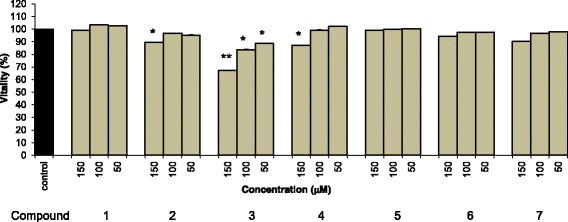
Cytotoxicity of compounds 1–7 isolated from *Crepis lacera* are as follows: crepiside D (**1**), 8-epidesacylcynaropicrin-3-*O*-*β*-glucopyranoside (**2**), 8-epigrosheimin (**3**), 8-*β*-hydroxydehydrozaluzanin C (**4**), 11-dehydrocrepiside D (**5)**, *p-*hydroxy-benzyl 7-O- β-glucopyranoside (**6**), and pynoresynol (**7**). MDBK cells were incubated with each compound for 48 h. Cell viability was assessed by MTT assay. The results represent mean values of three independent experiments ± standard deviations (SD). *P < 0.05 vs control (cells incubated with vehicle only); **P < 0.01 vs control

This in vitro study undertaken using MDBK cells to evaluate the possible cytotoxicity of the compounds isolated from the plant represents the first study on the effects of *Crepis spp.* in a ruminant cell model. The MTT assay results showed that no examined compounds significantly affected MDBK cell viability after 24 h of incubation (data not shown); however, three sesquiterpenes, 3-epidesacylcynaropicrin-8-O-β-glucopyranoside (**2**), 8-epigrosheimin (**3**) and 8-hydroxydehydrozaluzanin C (**4**), were significantly cytotoxic after 48 h (Fig. 6). All other assessed compounds were only weakly toxic or inactive under our experimental conditions.

The in vivo study was performed by administering *C. lacera* extract to rats. The control group, sacrificed after 24 and 48 h, showed histological features of normal renal, hepatic and pulmonary tissues. The animals that received 1 mg/kg bw of *Crepis lacera* extract and were sacrificed after 24 h showed no significant lesions in their liver, lungs or kidneys. By contrast, the presence of a few shrunken glomeruli and moderate signs of degenerated tubular epithelium characterized by small cytoplasmic vacuoles were observed mainly in the kidneys of animals sacrificed after 48 h of consuming the plant extract. Moderate signs of degeneration characterized by small cytoplasmic vacuoles were also observed in their livers. The rats that received 2 mg/kg bw of *Crepis lacera* extract and were sacrificed after 24 h presented large perivascular hemorrhages in the renal medulla, severe glomerular hemorrhages and presence of shrunken glomeruli, marked degenerative changes and necrotic tubular epithelia in both the distal and proximal renal tubules. Severe degeneration with large intra-cytoplasmic vacuoles and disrupted hepatic architecture was observed in the livers. The lungs showed numerous wide hemorrhagic lacunae with the appearance of flooding mixed with emphysematous areas. These lesions did not differ noticeably from those of the rats sacrificed after 48 h**.**

## Discussion

Fatal poisoning by toxic plants in domestic animals is common worldwide [8–11]. Often, such poisoning occurs during dry seasons due to a relative shortage of alternative forage.

Sudden unexplained deaths in many grazing animals is a frequent event in some areas of southern Italy (i.e., Basilicata and Campania), particularly during late spring and throughout the summer, causing severe economic loss. Often, the causes of these deaths are unexplained because the necropsies cannot be performed in time. Interestingly, the dead animals are often the strongest and healthiest in the herd. In addition, the number of intoxicated animals decreased when they moved to other pastures. This led farmers to hypothesize that the cause could be a poisonous plant growing in sections of the pastures where only the stronger sheep could climb, or the stronger sheep arrived before the weaker animals and ingested more of the plant. *Crepis lacera*, which grows at altitudes of 700 to 1200 m, corresponds to these characteristics. In addition, the plant’s life cycle is characterized by two flowering periods: the first during spring and the second in early autumn. *C. lacera* shows properties common to many bitter herbs; therefore, grazing animals likely do not ingest this plant when other plants are available. However, domestic herbivores may ingest *Crepis lacera* during dry periods, when forage is lacking. We were contacted by farmers at the beginning of the dry season reporting the sudden deaths of many sheep, leading us to suspect *Crepis lacera* poisoning.

The current study describes sheep poisoning after pasture grazing. The on-site inspection revealed that *C. lacera* grew abundantly in the area, confirming it as a potential cause of death. Regarding the cardiovascular apparatus examination, the bradycardia described in both sheep likely resulted from an electrolyte imbalance and metabolic acidosis due to renal and hepatic injury. One sheep showed sporadic ventricular premature complexes. VPCs are ectopic impulses originating from an area distal to the His Purkinje system and are characterized by a QRS complex that differs morphologically on the ECG. Premature complexes of all origins generally indicate myocardial disease, but atrial premature complexes accompanying cases of gastrointestinal disease have been described in cattle [12]. Ventricular premature contractions related to myocardial lesions were also described in adult horses at rest and during exercise [13]. By contrast, Sudhakara Reddy et al. [14] suggested that ventricular premature complexes could be physiological cardiac arrhythmias. In our case, without histopathological signs of myocardial lesions, VPCs, as well as bradycardia, may be related to electrolyte imbalance and metabolic acidosis due to renal/hepatic injury or alternatively, VPCs could be physiological cardiac arrhythmias [14]. Little is known about *Crepis lacera* chemical compounds; thus, it is uncertain if this plant contains toxic substances. Scientific evidence of a relationship between *Crepis lacera* ingestion and death in domestic animals is lacking. Therefore, we conducted a phytochemical study to characterize some of *Crepis lacera*’s main compounds. In addition, we assessed the biologic activity of those isolated compounds in an in vitro model of ruminant kidney cells. To date, our in vitro study using MDBK cells to evaluate the possible toxicity of compounds isolated from *C. lacera* is the first study on the effects of *Crepis spp.* in ruminant cells. We found that three sesquiterpenes, 3-epidesacylcynaropicrin-8-*O*-β-glucopyranoside, 8-epigrosheimin and 8-hydroxydehydrozaluzanin C, exerted cytotoxic effects in MKBD cells after 48 h. These in vitro data corroborate the necropsy findings and may partially explain the mechanisms leading to the gross and histopathological lesions identified at necropsy in the fatally poisoned sheep kidneys. In addition, histological lesions observed in rats challenged with higher doses of toxic extracts, exhibited signs of acute *Crepis lacera* toxicity after 24 and 48 h, confirming the hepatic and renal tropism of the plant toxins.

## Conclusions

These results corroborate the hypothesis that *C. lacera* is dangerous to sheep when ingested in large amounts. This study describes the first evidence of *Crepis* spp. poisoning in sheep, which is of concern due to the increasing diffusion of this botanical species in many Mediterranean countries including Italy, France, Spain, and Greece. Further investigations are needed to better understand the toxicity of *Crepis lacera*, and molecular studies are necessary to characterize the mechanism of action of the plant’s toxic compounds. In addition, field studies are necessary to improve botanical knowledge of this plant species including its characteristic features, habitat, environmental growth conditions, diffusion and areas of infestation. Such information is necessary to manage livestock and define measures to prevent ruminant poisoning during grazing, which results in substantial animal production losses.

## Abbreviations

bw: Body weight
*C. lacera*: *Crepis lacera*
CHCl_3_: Chloroform
CHCl_3_-MeOH: Chloroform-methanol
DMEM: Dulbecco’s modified Eagle’s medium
ECG: Electrocardiograms
FCS: Fetal calf serum
MDBK: *Madin Darby Bovine Kidney*
MeOH: Methanol
OD: Optical density
po: Per os
RP: Reversed-phase
RT: Retention time
S.D.: Standard deviation
Sec: Seconds
